# Altered White Matter Integrity in the Congenital and Late Blind People

**DOI:** 10.1155/2013/128236

**Published:** 2013-04-18

**Authors:** Dawei Wang, Wen Qin, Yong Liu, Yunting Zhang, Tianzi Jiang, Chunshui Yu

**Affiliations:** ^1^Department of Radiology, Tianjin Medical University General Hospital, Tianjin 300052, China; ^2^Brainnetome Center, Institute of Automation, Chinese Academy of Sciences, Beijing 100190, China; ^3^National Laboratory of Pattern Recognition, Institute of Automation, Chinese Academy of Sciences, Beijing 100190, China

## Abstract

The blind subjects have experienced a series of brain structural and functional alterations due to the visual deprivation. It remains unclear as to whether white matter changes differ between blind subjects with visual deprivation before and after a critical developmental period. The present study offered a direct comparison in changes of white matter fractional anisotropy (FA) between congenital blind (CB) and late blind (LB) individuals. Twenty CB, 21 LB (blindness onset after 18 years old), and 40 sight control (SC) subjects were recruited. Both the tract-based spatial statistics (TBSS) and voxel-based analysis (VBA) showed lower FA in the bilateral optic radiations in both blind groups, suggesting that the loss of white matter integrity was the prominent hallmark in the blind people. The LB group showed more extensive white matter impairment than the CB group, indicating the mechanisms of white matter FA changes are different between the CB and LB groups. Using a loose threshold, a trend of an increased FA was found in the bilateral corticospinal tracts in the LB but with a smaller spatial extent relative to the CB. These results suggest that white matter FA changes in the blind subjects are the reflection of multiple mechanisms, including the axonal degeneration, deafferentation, and plasticity.

## 1. Introduction

The blind subjects, irrespective of the age of onset, have experienced a series of structural and functional alterations, and they have to make major adjustments to interact effectively with the environment. Numerous functional magnetic resonance imaging (fMRI) studies have revealed that the occipital cortex of the blind subjects shifts its function to process tactile [1] and auditory information [2] and to engage in many higher-level cognitive functions, such as language [3–8], memory [3], and mental imagery [9–11]. Structural MRI studies have shown the decreased gray/white matter volume [12–14] but increased cortical thickness in the occipital cortex [15, 16].

During the past two decades, diffusion tensor imaging (DTI), as a noninvasive means, makes the* in vivo* evaluation of white matter integrity possible using the fractional anisotropy (FA) [17–19]. The decreased FA may represent the impairment in white matter integrity, in contrast, the increased FA may indicate the increase in white matter integrity [17–19]. Different DTI analytic methods have shown that congenitally blind (CB) or early blind (EB) subjects had atrophy [20] or decreased white matter integrity [21] in the optic radiation (OR), reduced efficiency of the brain anatomical network [22], and increased white matter integrity in the corticospinal tract (CST) [23]. However, inconsistent findings have been reported in late blind (LB) subjects. No significant decreased white matter integrity in the ORs has been reported in a voxel-based analysis (VBA) in a group of six LB individuals [24] and in a diffusion tensor tractography (DTT) based analysis in LB subjects [25]. In contrast, significant atrophy was found in the visual cortices in both the LB and EB subjects [13].

In the present study, we applied both the tract-based spatial statistics (TBSS) and VBA methods in 20 CB, 21 LB, and 40 SC subjects to address the following questions: (1) does the white matter damage in the CB differ from that in the LB? (2) Does the white matter plasticity differ between subjects who blinded before and after the critical developmental period?

## 2. Materials and Methods

### 2.1. Subjects

Twenty CB (13 males, mean age 26.6 ± 5.0 years, age range: 20–39 years), 21 LB (16 males, mean age 33.1 ± 7.1 years, age range: 23–46 years) and 40 sighted controls (SC) (22 males, mean age 31.8 ± 7.2 years, age range: 21–47 years), participated in this experiment after giving written informed consent in accordance with the Medical Research Ethics Committee of Tianjin Medical University. All CB subjects had lost their sight since birth. None of the CB subjects had a history of normal vision, and none had memories of visual experience. LB subjects had lost their sight after 18 years old, and they had no visible brain lesions on conventional brain MR images. 

### 2.2. MRI Data Acquisition

DTI data were obtained using a 3.0-Tesla MR scanner (Trio Tim system; Siemens, Erlangen, Germany) with a 12-channel head coil. Tight but comfortable foam padding was used to minimize head movement. Diffusion weighted images were acquired employing a single-shot echo planar imaging (EPI) sequence in alignment with the anterior-posterior commissural plane. The integral parallel acquisition technique (iPAT) was used with an acceleration factor of 2, which can reduce image distortion from susceptibility artifacts. Diffusion sensitizing gradients were applied along 30 nonlinear directions (*b* = 1000 s/mm^2^) together with an acquisition without diffusion weighting (*b* = 0 s/mm^2^). The imaging parameters were 45 continuous axial slices with a slice thickness of 3 mm and no gap, repetition time/echo time (TR/TE) = 6000/90 ms, field of view (FOV) =256 mm × 256 mm, and matrix size = 128 × 128. The reconstruction matrix was 256 × 256, resulting in a voxel dimension of 1 mm × 1 mm × 3 mm. The acquisitions were repeated 2 times to improve the signal-to-noise ratio.

### 2.3. Tract-Based Spatial Statistics

All diffusion-weighted images were visually inspected by two radiologists for apparent artifacts due to subject motion and instrument malfunction. The Eddy-current-induced distortion and motion artifact in the DTI dataset were corrected by applying affine alignment of each diffusion-weighted image to the *b* = 0 image using FMRIB's diffusion toolbox (http://www.fmrib.ox.ac.uk/fsl, FSL 4.0) [26]. A diffusion tensor model was fit to the set of diffusion-weighted images before calculating FA maps for each subject. 

The following steps were adopted for the TBSS analysis [27]. All subjects' FA images were aligned to a template of the averaged FA images (FMRIB-58) in Montreal Neurological Institute (MNI) space using a nonlinear registration algorithm implemented in FNIRT (FMRIB's nonlinear registration Tool) [28]. After transformation into MNI space, a mean FA image was created and thinned to generate a mean FA skeleton of the white matter tracts. Each subject's aligned FA images were then projected onto the mean FA skeleton by filling the mean FA skeleton with FA values from the nearest relevant tract center, which was achieved by searching perpendicular to the local skeleton structure for maximum value. This second local coregistration step can alleviate the malalignment of diffusion-weighted images during the former registration step.

Voxel-wise statistical analysis across subjects on the skeleton space was carried out using a permutation-based inference tool for nonparametric statistic (“randomize”, part of FSL). In the present study, we enumerated all three combinations of comparison groups (specifically, the CB versus SC, LB versus SC, and the CB versus LB) and tested the differences in white matter integrity for each combination. Voxel-wise group comparisons were performed using a general linear model for each combination with controlling for age and gender. The mean FA skeleton was used as a mask (thresholded at a mean FA value of 0.2 to include only major fiber bundles and exclude peripheral tracts with significant intersubject variability), and the number of permutations was set to 5000. The significance threshold for intergroup differences was set at *P* < 0.05 after correcting for family wise error (FWE) using the threshold-free cluster enhancement (TFCE) option in permutation-testing tool in FSL. 

### 2.4. Voxel-Based Analysis of FA Images

Utilizing SPM8 package, each subject's *b* = 0 images were first normalized to the EPI template in MNI space. The normalization consisted of a 12-iteration linear transformation and a nonlinear transformation with 7 × 8 × 7 basis functions. Parameters from this transformation were then applied to each subject's FA images, and then resampled the volume into a voxel size of 2 mm × 2 mm × 2 mm. Further, each normalized FA image was spatially smoothed by an isotropic full-width at the half maximum Gaussian kernel of 8 mm × 8 mm × 8 mm to reduce the effect of misregistration in spatial normalization [29, 30]. We performed two-sample *t*-tests for each combination of groups on the normalized FA images in a voxel-based manner after controlling for age and gender. The significant threshold value was set at *P* < 0.01 after correcting for false discovery rate (FDR) and a cluster size of >30 voxels.

### 2.5. Correlation Analyses

To address if the reduced FA in the visual and nonvisual regions in the LB are correlated with the chronological age, age at blindness onset, and duration of blindness in these LB subjects, region-of-interest- (ROI-) based correlation analysis was performed. The ROIs were defined as the six spheroid ROIs with a radius of 6 mm and centered at the peak locations of regions that exhibited significant reduced FA in the LB subjects compared to the SC. These spheroid ROIs were then multiplied by the voxels with significantly reduced FA in white matter skeleton to ensure that all remainder voxels of the ROIs had reduced FA. After the extraction of each ROI, the mean FA value of the ROI was calculated. Finally, correlations were calculated between the FA of each ROI and the chronological age, age at blindness onset, and duration of blindness in these LB subjects using the Statistical Package for the Social Sciences version 16.0 (SPSS, Chicago, IL, USA). Using the same method, we also investigated if the reduced FA in ORs in the CB is correlated with the chronological age that is equal to the duration of blindness in the CB subjects.

## 3. Results

### 3.1. Demographic Statistics

The demographic information is shown in Table 1. Significant group difference was found in age (*F* = 5.63,  *P* = 0.005). Although no significant group difference was found in gender (*χ* ^2^ = 2.70,  *P* = 0.26), subtle difference existed in ratio of males/total subjects among the three groups (0.65 in CB; 0.76 in LB; and 0.55 in SC). Consequently, individuals' age and gender were treated as a covariate of no interest in both the TBSS and VBA statistics.

### 3.2. TBSS Analysis

**Figure 1:**
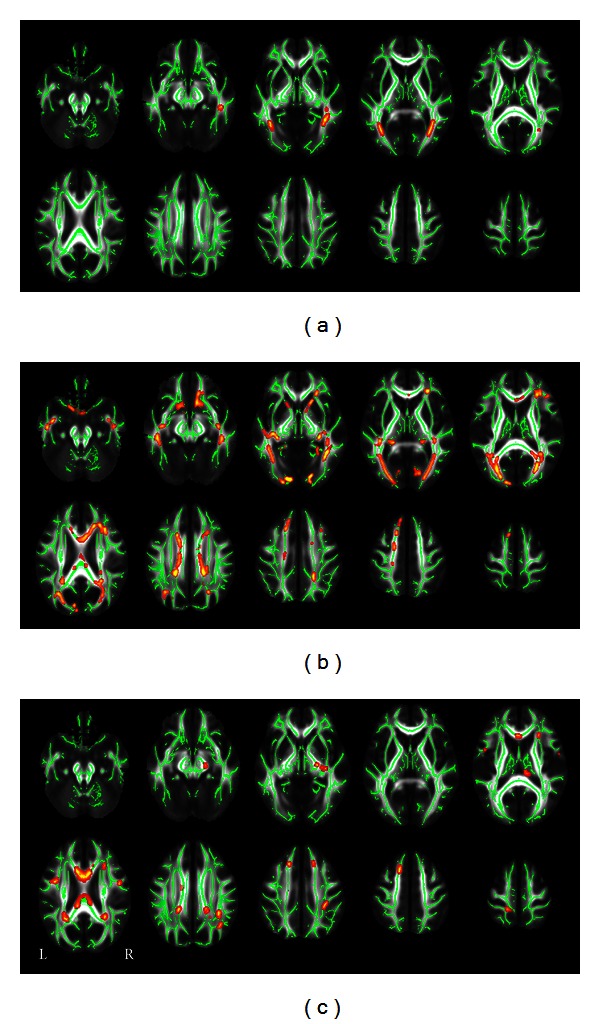
TBSS shows FA differences between three combinations of groups ((a) CB < SC; (b) LB < SC; (c) CB > LB). Green represents mean FA skeleton of all participants; red represents regions with significant group differences (*P* < 0.05, FWE corrected). CB: congenitally blind; FA: fractional anisotropy; SC: sighted control; TBSS: tract-based spatial statistics; L: left; LB: late blind; and R: right.

**Figure 2:**
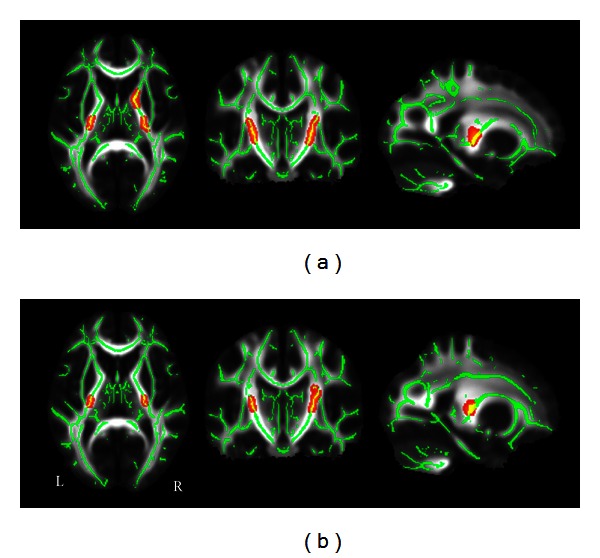
TBSS shows increased white matter FA in CB (a) and LB (b) compared to the SC using a loose thfreshold. Green represents mean FA skeleton of all participants; red color represents regions with significant increased FA in each of the two blind groups (*P* < 0.05, uncorrected). CB: congenitally blind; FA: fractional anisotropy; SC: sighted control; TBSS: tract-based spatial statistics; L: left; LB: late blind; and R: right.

Compared with the SC group, the CB group showed significantly lower FA in the ORs bilaterally (*P* < 0.05, FWE corrected) (Figure 1(a)), whereas the LB group showed significantly lower FA in the bilateral ORs, corpus callosum, anterior thalamic radiations, and frontal and parietal white matter regions (*P* < 0.05, FWE corrected) (Figure 1(b)). The regions showing lower FA were more extensive in the LB than in the CB group. We then directly compared the CB and LB groups and found that the CB group showed higher FA mainly in corpus callosum, right thalamus, and frontal and parietal white matter regions relative to the LB group (*P* < 0.05, FWE corrected) (Figure 1(c)). No significantly increased FA was found in either the CB or the LB group when compared to the SC group (*P* < 0.05, FWE corrected). Because a previous DTT study has reported increased FA in the CSTs in EB subjects [23], we want to test whether such phenomenon was present in the present dataset. Consequently, we used a loose threshold (*P* < 0.05, uncorrected) and found that the CB group showed increase FA in the bilateral CSTs and right anterior thalamic radiation when compared with the SC (Figure 2(a)), and increased FA in the bilateral CSTs was also shown in the LB group relative to the SC group (Figure 2(b)).

### 3.3. VBA of FA Images

**Figure 3:**
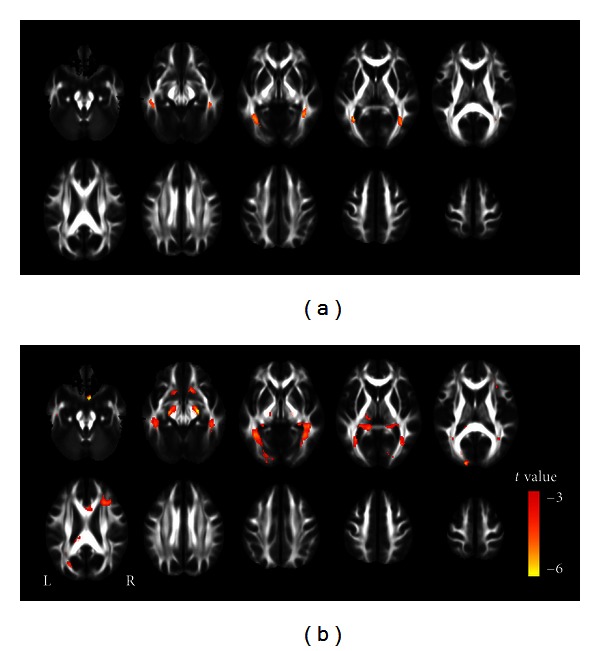
FA differences between the CB or LB and SC groups using a VBA method. The CB subjects show decreased FA in the bilateral optic radiations compared to the SC subjects (a). The LB subjects show decreased FA in multiple regions than the SC subjects (b). Red represents regions with significant decreased FA in each of the two blind groups (*P* < 0.01, FDR corrected). CB: congenitally blind; FA: fractional anisotropy; SC: sighted control; L: left; LB: late blind; R: right; and VBA: voxel-based analysis.

VBA revealed similar results as what TBSS analysis had found, showing significantly lower FA in ORs bilaterally in both the CB and LB individuals, and the regions showing lower FA were more extensive in the LB than in the CB group, which mainly consisted of the corpus callosum, right thalamus, and frontal and parietal white matter regions (*P* < 0.01, FDR corrected) (Figures 3(a) and 3(b)).

### 3.4. Correlation Analyses

**Figure 4:**
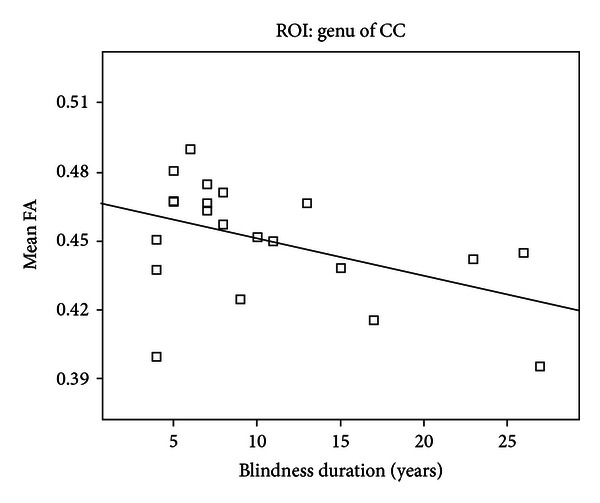
A trend of correlation between the FA value of the genu of the corpus callosum and duration of blindness in the LB (*P* < 0.05). CC: corpus callosum; FA: fractional anisotropy; and LB: late blind.

In the CB subjects, we did not find any significant correlations between the FA values of the ROIs of the left (*r* = −0.26,  *P* = 0.27) and right (*r* = −0.21,  *P* = 0.38) ORs and the duration of blindness. In the LB, the FA values of all six ROIs were not significantly correlated with the chronological ages and ages at blindness onset (Table 2). Although the FA value of corpus callosum showed correlation with the duration of blindness (*r* = −0.47,  *P* = 0.031) (Figure 4), this correlation can survive neither after correction for multiple comparisons nor after correction for the chronological ages. Moreover, the FA values of other five ROIs were not correlated with the duration of blindness (Table 2).

## 4. Discussion

### 4.1. Methological Consideration

The VBA is a relatively simple exploratory method to detect the white matter FA changes between groups [31, 32]. However, this method suffers from the problem of alignment of FA images from different subjects and the arbitrary choice of smoothing kernels [30]. The TBSS may partly avoid the two problems [27]; however, this method may miss the differences in the near-cortical fibers because the TBSS only focuses on the center skeleton of white matter tracts. The combination of these two methods may improve the reliability of our results. In this study, well-consistent results have been showed between the VBA and TBSS methods for the FA differences between groups.

### 4.2. White Matter Impairment in CB and LB

Compared with the SC group, the CB group showed decreased FA only in the ORs bilaterally, whereas the LB group showed reduced FA in a more extensive spatial extent, including the bilateral ORs, corpus callosum, anterior thalamic radiations, and frontal and parietal white matter regions. These findings suggest that the mechanisms of white matter FA changes may differ between the CB and LB groups. The white matter integrity in the LB may be affected through mechanisms of axonal degeneration secondary to the impairment of anterior portion of the visual neural pathway and secondary changes in neural circuits whose activity depends largely on visual stimuli due to the visual deprivation (deafferentation). Both mechanisms may result in decreased white matter FA value. The axonal degeneration mechanism may explain the decreased FA in the OR in the LB subjects because this mechanism has been used to explain the decreased white matter integrity in the OR in patients with lesions in the anterior portion of the visual pathway, such as optic neuritis [33, 34] and neuromyelitis optica [35–37]. 

The deafferentation mechanism may help to understand white matter integrity impairments in nonvisual white matter regions in the LB. Brain structural impairments including white matter integrity impairment due to the deprivation or the lack of afferent input (deafferentation) have been well investigated. For instance, reduced FA values in the corpus callosum [38] and decreased gray matter volume of the posterolateral thalamus [39] were found in patients with amputation (somatosensory deafferentation). Animal studies have showed that the brainstem and thalamic ventral posterior nuclei were severe atrophy due to upper-limb deafferentation [40]. Recently, Bock and coauthors investigated structural changes in the bilaterally enucleated ferrets at postnatal day 7 (P7; early visual deprivation) and P20 (late visual deprivation) and found callosal connection damage in P7 ferrets and visual cortex atrophy in both P7 and P20 ferrets [41]. Moreover, patients with glaucoma and amblyopia could be regarded as partial deafferentation models of the visual system, the reduced FA in the ORs has been observed in these patients [42, 43]. All these findings suggest that the brain white matter integrity could be affected by the deafferentation of the relevant sensory input even in adulthood. However, our findings in the LB are not consistent with two previous studies on the white matter integrity changes in the LB [24, 25]. One study showed optic pathways remaining intact in a group of six LB individuals [24], while the relatively small sample size in that study might be less sensitive to find group differences. Another study found that the white matter integrity of the ORs remained normal in acquired blindness using a ROI analysis [25]; however, the ROI of the OR in that study included non-OR tissues [25], which may explain the lack of difference in the white matter integrity in the OR between the two groups. Moreover, the impairment of white matter integrity in the ORs were found even in patients with amblyopia [42] and glaucoma [43] who were partial deafferentation; there should exist white matter abnormality in the ORs in the LB subjects who were completely deafferentation.

White matter impairment in ORs in CB or EB individuals has been well investigated by several studies [20, 21, 44]. Shimony and coauthors reported a higher MD and lower anisotropy values in the OR in 5 EB subjects when compared with the SC [20]. Similarly, Noppeney and colleagues found significantly reduced white matter density in the OR in the EB subjects relative to the SC [44]. Recently, Shu and coauthors used both the VBA and TBSS methods to reveal the reduced white matter integrity in the OR in the EB [21]. As in the LB, both the mechanisms of axonal degeneration [45] and deafferentation may also result in the white matter integrity changes in the OR in the CB or EB. Additionally, developmental immaturity [46–48] due to the visual deprivation before the critical developmental period may be also implicated in the mechanism of the white matter integrity changes in the OR in the CB or EB. 

In the present study, we found that the LB showed more extensive white matter impairments relative to the CB. The less impairment of white matter integrity in CB suggests that the CB individuals may benefit more from the white matter plasticity or compensatory adaptations due to a larger potential of plasticity in the CB than in the LB. In the CB who undergoes an early deprivation of visual input, plastic changes could occur in the auditory, sensory and motor cortices [15, 49, 50], even in higher-level cognitive regions such as prefrontal and frontal regions [13]. Although visual input is absent in CB, input from other sensory modalities such as auditory, tactile, and olfactory information may compensate to maintain the white matter development that mainly depends on visual input in the SC, especially during the critical developmental period. Therefore, in the CB individuals, the white matter plasticity during the critical developmental period might compensate for the impairment resulting from the deafferentation of the visual information, which might account for why the CB subjects show fewer regions with white matter impairments relative to the LB.

### 4.3. White Matter Plasticity in CB and LB

In this study, using a loose threshold, we found that the CB showed increased FA in CST than the SC, which was similar with what we had found previously [23], suggesting that experience-dependent plasticity may thus induce an increase in the FA of the CST in the CB. It is generally agreed that CB or EB individuals, who lost their sight early in life, benefit from plastic changes in response to visual deprivation [23, 42], and the blind people needs more practice to perform the same routine activities of the sighted subjects, which would increase myelination of relevant axons. Interestedly, increased FA in CSTs was also found in the LB individuals whose visual system has been well developed, suggesting that the potential of CST plasticity may persist into the adulthood, which hypothesis has been confirmed in stroke patients [49, 50]. Consistent with our findings, several studies have shown the plastic changes in the brain in the LB individuals [4, 13, 51, 52]. However, the plastic changes in the LB were not as remarkable as those for the EB, which supports the concept that the potential of brain plasticity may decrease with age, although brain plasticity may extend into adulthood or even into aged-people. It should be noted that our findings were only at a loose threshold and validation should be done in future studies.

### 4.4. Correlation Analyses

In the CB subjects, we did not find any significant correlations between the FA values of the ROIs of the left and right ORs and the duration of blindness. This finding appears to be inconsistent with a previous study that showed the ORs were continued atrophy in adult years for EB [51]. Several differences between the two studies may account for the discrepancy: (1) different measures: white matter volume in the previous study [51] and FA in our study; (2) different onset ages of blindness: within 6 years old in the previous study [51] and congenital blindness in our study; and (3) different chronological ages: 38.5–58.2 years old in the previous study [51] and 20–39 years old in our study. In our study, only blindness itself affects the white matter integrity in CB subjects, whereas both the blindness and aging may affect white matter volume in the previous study [51]. In the LB, the FA values of all six ROIs were not significantly correlated with the chronological ages and ages at blindness onset. Although the FA value of corpus callosum showed correlation with the duration of blindness, this correlation can survive neither after correction for multiple comparisons nor after correction for the chronological ages. Moreover, the FA values of other five ROIs were not correlated with the duration of blindness. These findings suggest that most of the FA values of these ROIs were not correlated with the chronological age, age at blindness onset, and duration of blindness in the LB subjects. The trend of correlation between the FA value of the genu of the corpus callosum and the duration of blindness may be explained by aging effect on white matter integrity because several studies have shown that the white matter in the genu of the corpus callosum is especially vulnerable to age-related microstructural changes [52, 53].

## 5. Conclusion

In this study, we offered a direct comparison between the CB and LB individuals to investigate how white matter integrity was changed in these two blind groups using both TBSS and VBA methods in a whole brain manner. We found that both the CB and LB groups showed white matter impairment, suggesting that the loss of white matter integrity was the prominent hallmark in the blind people. The LB group showed more extensive white matter impairment than the CB group, indicating that the mechanisms of white matter integrity changes are different between the CB and LB groups.

## Figures and Tables

**Table 1:** Demographic information of participants.

	CB	LB	SC	*P* value
Number of subjects	20	21	40	
Age (years)	26.6 (5.0)	33.1 (7.1)	31.8 (7.2)	0.005
Age of onset	0	22.6 (4.5)	None	
Gender (male/female)	13/7	16/5	22/18	0.26

Data is means (SD); CB: congenitally blind; SC: sighted control; and LB: late blind.

**Table 2:** Correlations between FA values of each ROI and duration of blindness, age of onset, and chronological age in the LB.

ROI	MNI coordinates	Blindness duration		Age of onset		Chronological age	
		*r *	*P* value	*r *	*P* value	*r *	*P* value
L_OR	−24, −80, 1	0.14	−0.041	0.86	0.56	0.11	0.64
R_OR	30, 72, 4	−0.25	−0.10	0.67	0.27	−0.32	0.16
Genu of CC	6, 13, 22	−0.47	0.086	0.71	**0.031**	−0.42	0.057
ATR	16, 17, −3	−0.41	0.21	0.37	0.070	−0.28	0.22
aIFOF	28, 33, 8	−0.042	−0.041	0.86	0.86	−0.068	0.77
pSLF	20, −41, 39	−0.051	0.20	0.93	0.83	−0.039	0.87

aIFOF: anterior inferior frontal occipital fasciculus; ATR: anterior thalamic radiation; CC: corpus callosum; FA: fractional anisotropy; OR: optic radiation; pSLF: posterior superior longitudinal fasciculus; and *r*: correlation coefficient.
